# QuickStats

**Published:** 2014-01-24

**Authors:** FROM THE NATIONAL CENTER FOR HEALTH STATISTICS

**Figure:**
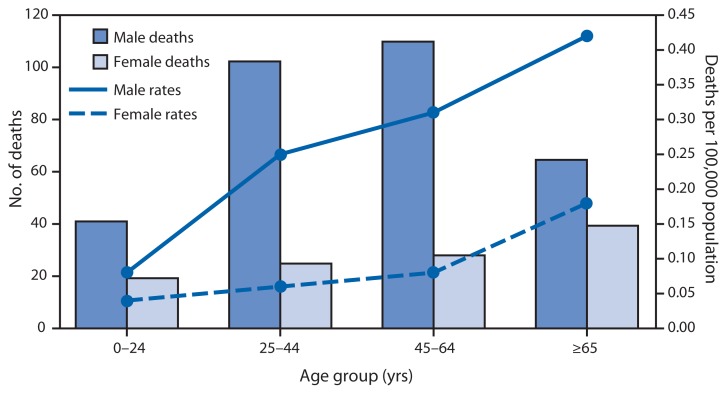
Average Annual Number of Deaths and Death Rates from Unintentional, Non–Fire-Related Carbon Monoxide Poisoning,*^†^ by Sex and Age Group — United States, 1999–2010 * Unintentional, non–fire-related carbon monoxide poisoning is defined both as 1) accidental poisoning by and exposure to gases or vapors (code X47) listed as the underlying cause, and 2) toxic effect of carbon monoxide (code T58) listed as the contributing cause, according to the *International Classification of Diseases, 10th Revision*. All deaths caused by intentional exposure (X67), exposure of undetermined intent (Y17), or fire-related exposure to carbon monoxide (codes X00–X09, X76, X97, and Y26) were excluded. ^†^ Deaths are 12-year annual averages, and death rates are per 100,000 12-year annual average population.

During 1999–2010, a total of 5,149 deaths from unintentional carbon monoxide poisoning occurred in the United States, an average of 430 deaths per year. The average annual death rate from carbon monoxide poisoning for males (0.22 per 100,000 population) was more than three times higher than that for females (0.07). The death rates were highest among those aged ≥65 years for males (0.42) and females (0.18). The rates were the lowest for males (0.08) and females (0.04) aged <25 years.

**Source:** National Vital Statistics System. Mortality public use data files, 1999–2010. Available at http://www.cdc.gov/nchs/data_access/vitalstatsonline.htm.

**Reported by:** Jiaquan Xu, MD, jiaquanxu@cdc.gov, 301-458-4086.

